# Stress‐induced peak (but not resting) metabolism correlates with mating display intensity in male guppies

**DOI:** 10.1002/ece3.2373

**Published:** 2016-08-18

**Authors:** Peter A. Biro, Kerry V. Fanson, Francesca Santostefano

**Affiliations:** ^1^ School of Life and Environmental SciencesDeakin UniversityGeelongVic.3216Australia; ^2^ Evolutionary Ecology of Variation GroupMax Planck Institute for OrnithologySeewiesenGermany

**Keywords:** Consistent individual differences, fish, habituation, metabolism, personality

## Abstract

Recent empirical and conceptual papers have highlighted the potential for metabolism to act as a proximate mechanism for behavior that could explain animal personality (consistency over time). Under this hypothesis, individuals with consistently high levels of behavioral activity should also have high resting metabolic rate (RMR) as it can reflect capacity to process food and generate energy. We tested for the predicted positive covariance between RMR and three behaviors that differ in energy demands in 30 male guppies, using multivariate mixed models; we repeatedly measured their activity (10 times each), courtship displays (nine times), voracity (10 times), and metabolism (four‐times). Resting metabolic rate (measured overnight in respirometry trials) did not consistently differ among males, whereas initial peak metabolism measured during those same trials (*R *=* *0.42), and all behaviors were repeatable (*R *=* *0.33–0.51). RMR declined over time suggesting habituation to the protocol, whereas peak metabolism did not. Initial peak metabolism was negatively correlated with courtship display intensity, and voracity was positively correlated with activity, but all other among‐individual correlations were not significant. We conclude that RMR does not provide a proximate explanation for consistent individual differences in behavior in male guppies, and therefore the potential for independent evolution of these physiological and behavioral traits seems possible. Finally, we identify peak metabolism as a potential measure of the stress response to confinement, which highlights the value of considering various aspects of metabolic rates recording during respirometry trials.

## Introduction

At present, we still do not have a strong understanding of the proximate physiological mechanisms that might facilitate or constrain behavioral variation among individuals. Metabolism has recently attracted considerable interest as a proximate factor influencing consistent individual differences in behavior (Careau et al. 2008; Biro and Stamps 2010; Réale et al. 2010; Burton et al. 2011). Energy is required to fuel all life processes, and if individuals vary in their ability to generate energy (i.e., ATP), this could affect differences in the expression of behaviors that consume energy (e.g., courtship, aggression) and those that support energy production (e.g., foraging activity, aggressive defense of food (Metcalfe et al. 1995; Careau et al. 2008; see review in Biro and Stamps 2010). In other words, if metabolism reflects energetic capacity, individuals with consistently higher metabolism should also express consistently higher levels of energetically costly behaviors.

There is evidence to support the idea that resting metabolic rate (RMR) reflects the costs of running energetically expensive organs that support energy expenditure on a sustained basis (Daan et al. 1990; Ricklefs et al. 1996; see also reviews in Careau et al. 2008; and in Biro and Stamps 2010). On this basis, individuals with higher RMR are expected to be able to engage in energetically costly activities to a greater extent than those with low RMR. Thus, if RMR is consistent over time (repeatable), then we can also expect consistent individual differences in levels of behavioral activity that possess a significant energy cost (Careau et al. 2008; Biro and Stamps 2010). We might also expect that the strength of correlations between metabolism and behavior and the probability of detecting them to be higher for activities with a particularly high energetic cost (Biro and Stamps 2010).

If metabolism acts as a constraint on behavioral variation among individuals, we might also expect that different behaviors with different functions might covary with one another to form a “behavioral syndrome” (Biro and Stamps 2008, 2010; Sih and Bell 2008; Réale et al. 2010; Burton et al. 2011). Apart from the potential for metabolism to act as a proximate constraint on behavioral variation, context‐dependent trade‐offs between metabolism and risks of starvation or predation are thought to maintain individual differences in energetics and behavior within populations (Careau et al. 2008; Biro and Stamps 2010; Réale et al. 2010; Burton et al. 2011). For example, individuals with higher metabolism perform well when food resources are abundant, but not when scarce (Reid et al. 2011, 2012), and can suffer higher predation rates (Artacho and Nespolo 2009).

Despite the recent interest in metabolism as a possible proximate mechanism of behavioral variation, there is still limited empirical evidence at the among‐individual (intraspecific) level. Some recent studies have indeed found a positive correlation between metabolic rate and behavior (e.g., Metcalfe et al. 1995; Huntingford et al. 2010; Careau et al. 2011; Martins et al. 2011), whereas others have failed to find a significant relationship (Timonin et al. 2011; Le Galliard et al. 2013; Mathot et al. 2013; Merritt et al. 2013; Gifford et al. 2014; see also reviews in Biro and Stamps 2010; and Careau and Garland 2012). Of the studies that have found a relationship, it is often fairly weak and/or variable across contexts or groups (Lantová et al. 2011; Reid et al. 2011, 2012; Killen et al. 2012; Bouwhuis et al. 2013; Pang et al. 2015). Variable or weak associations suggest either a context‐, species‐, or behavior‐dependent nature of the associations; they may also be explained (at least in part) by limited statistical power associated with low sample sizes.

Given the low repeatability (*ca*. 0.4 on average) of metabolic and behavioral traits (Nespolo and Franco 2007; Bell et al. 2009; White et al. 2013), it is particularly important to have multiple repeated measures per individual, otherwise estimates of individual values are imprecise, and phenotypic correlations between traits become biased toward zero (Adolph and Hardin 2007). Indeed, most studies do not have more than two repeated measures per individual for metabolism or behavior, making it difficult to detect among‐individual correlations for traits with low repeatability (Adolph and Hardin 2007; Nespolo and Franco 2007; Bell et al. 2009; Wolak et al. 2012; Beckmann and Biro 2013; Biro and Stamps 2015). Thus, it is possible that some of the null and inconsistent correlations observed across studies are due to issues surrounding low statistical power. This highlights a need for more powerful studies to help resolve the equivocal nature of relationships between physiological and behavioral traits that exist in the literature.

Here, we investigated the among‐individual covariance between metabolism and behaviors in 30 male guppies (*Poecilia reticulata*), measured repeatedly over time (4 and 10 times each, respectively). We specifically tested the prediction that RMR should be positively correlated with behaviors with a significant energy cost, under the assumption that it reflects the capacity to generate energy (Careau et al. 2008; Biro and Stamps 2010). Given that the strength of any correlation between metabolism and behavior should depend upon the energetic costs of that behavior (Biro and Stamps 2010), we repeatedly measured behaviors that reflect different energy requirements: spontaneous activity with an assumed lower cost, and the frequency of courtship displays (in the presence of a female) with an assumed higher cost (Magurran et al. 1995; Magellan and Magurran 2007). We also repeatedly measured voracity (latency to feed when food is provided) in order to make inferences about hunger and food intake rates, under the expectation that higher energy output is supported on a sustained basis by increased energy intake together with higher RMR (Careau et al. 2008; Biro and Stamps 2010). In addition to RMR, we considered other metabolic measures that could be extracted from our respirometry trials that might provide additional insights, such as maximum MR that might provide information about the stress‐induced reaction of being forced into confined respirometry chambers (Careau et al. 2008). In a much broader context, our study aims to understand the causes of among‐individual variation in metabolic rates and in behavioral traits, and their covariance, as individual variation is the material upon which selection acts. If these traits covary, then selection on any one trait could lead to correlated selection on others, impeding independent trait evolution.

## Methods

### Study animals and housing

Guppies used in this experiment descended from individuals caught in 2009 in Alligator Creek, Bowling Green Bay National Park, Queensland (Queensland permit number WITK07655010). Fish were maintained in several large (170 L) stock tanks, each comprised of approximately 200 individuals (mixed age, mixed sex).

We used 30 sexually mature males selected randomly from stock tanks. Males were housed individually in 3‐L (25 × 15 × 10 cm) tanks. Each tank had a thin layer of brown natural gravel (3 mm diameter), an air stone for aeration, and opaque window covering on two sides to ensure fish could not see each other. Fish were fed once a day with commercial flake food and maintained at 24 ± 1°C under a 12:12 h light:dark photoperiod. Domestic water was dechlorinated with Water Purifier (Aquasonic, Port Macquarie, NSW, Australia) and treated with Hardness‐Up (Aquasonic) in order to raise hardness, and raise pH to 7.6. A small amount of sea salt was added to generate detectable salinity. We changed 60% of the aquaria water once per week. Research adhered to animal ethics guidelines (Deakin University permit #A93‐2011).

### Experiment overview

Observations of behavior and metabolism occurred in April and May 2012, and all behavioral observations were conducted by a single observer (FS). Following recent suggestions (Biro 2012), behavioral observations were conducted in the male's home tank after 1 week of acclimation. During the first 2 weeks of observations, we measured voracity and activity, always in this order, 10 times each on all 30 individuals. One week later, we started measuring MR, followed by courtship (2 days afterward), on a subset of 24 individuals. We repeated the alternating MR – courtship cycle three times, and measured MR once more at the end. Because only six fish could be measured for MR each day (details below), we divided the 24 males in four groups of six and tested them on different days. We therefore obtained four measurements of MR and nine (three per day) of courtship per individual. Behavioral observations were made from approximately 1 m away, and slow and careful movements did not appear to disturb the fish.

### Behavioral observations

Voracity was measured as the males’ latency to take the first bite of food during a feeding trial. Using our standard feeding procedure (also used during the week of acclimation), fish were fed between 9:30 and 11:00 each day using a transfer pipette to add a few drops of crushed flake food that had been mixed with water. Feeding latency was capped at 120 sec because the majority of the fish had initiated feeding in this time as determined from pilots, and because the flake food settles out of the water column and into the gravel after this time interval making it difficult to observe the first bite of food; there were 45 instances where we observed values of 120 sec. Routine (spontaneous) activity was measured once per day (between 10:00 and 17:00). Observations took place at least 60 min after feeding and no other stimulus was applied; therefore, measurements reflect routine activity level. To quantify activity, we drew a grid (4 × 4 cm squares) on the side of the tank and counted the number of lines crossed within 3 min. A fish was judged to have crossed a line once its head and pectoral fins had crossed the line.

Courtship trials began approximately 1 week after activity observations were completed. Each male was paired with a different (unique) female on three different occasions, each approximately 1 week apart. Pairs were observed at three time points during the trial: immediately after the female was introduced, 1 h after introduction, and 4 h after introduction, yielding three repeated measures of courtship per male per occasion, for a total of nine observations per male. We recorded the total number of sigmoid displays performed in 10 min as our measure of courtship intensity (Magurran et al. 1995; Magellan and Magurran 2007). Stimulus females were randomly chosen from a stock tank with a female‐biased sex ratio (~90% females) and were never used more than once. Six males were tested each day, by staggering the introduction of females to tanks. If the female did not move and was completely unresponsive during an observation session (32%), that session was excluded from analysis.

### Measurement of metabolism

**Figure 1:**
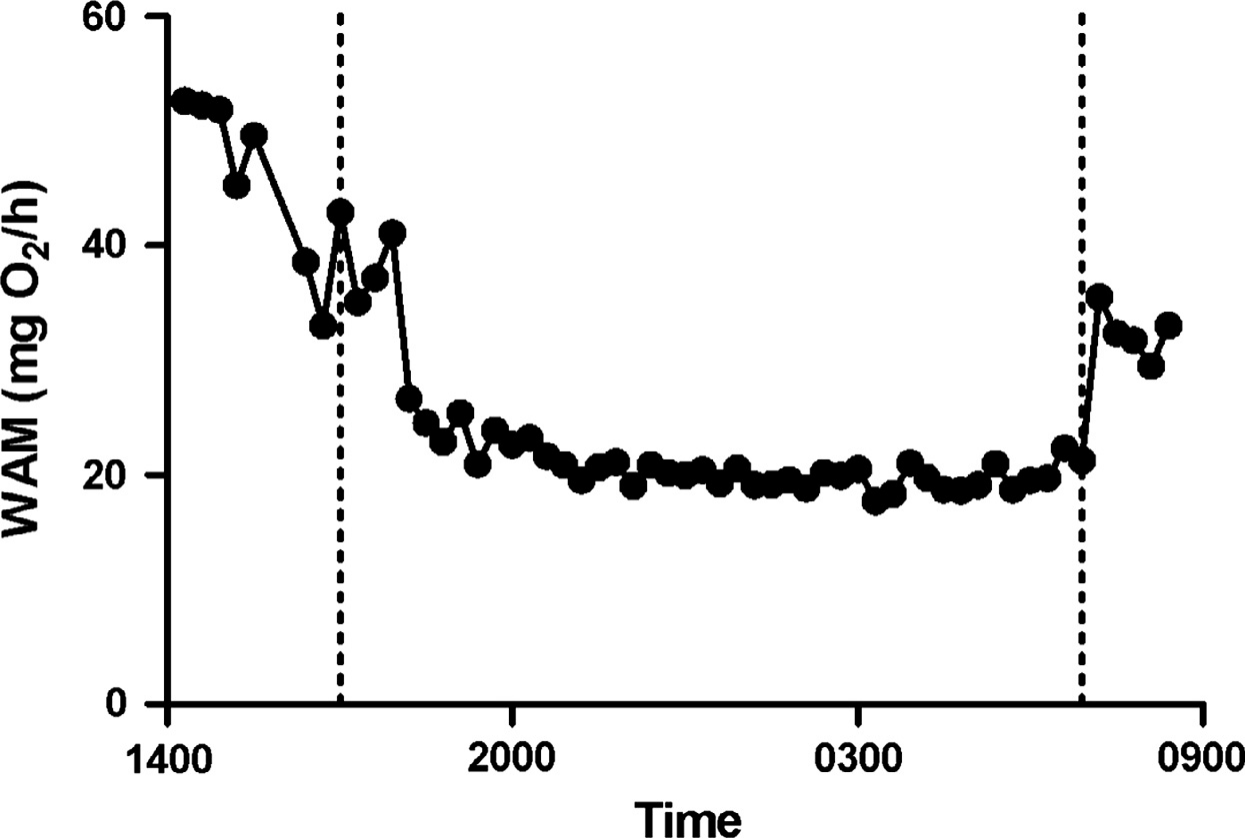
Whole‐animal metabolic rate (WAM) of a representative individual male guppy, during a single respirometry trial. Each dot represents oxygen consumption over an 8‐min measurement period. Vertical reference lines indicate sunset (17:00) and sunrise (07:30).

We measured six fish at one time, and those same individuals were exposed to a courtship assay 2 days later, before another respirometry trial 3 days after that (see above). Most (*n* = 22) but not all males were measured four times each; two individuals were measured only three times each, because one jumped out of the aquarium and another died of unknown causes. Each respirometry trial began by fasting fish 36 h prior to transfer to respirometry chambers, where they remained undisturbed overnight, from late afternoon (14:00–16:00) until the following morning at 09:00 h when data collection was terminated (Fig. 1).

Oxygen consumption was measured using intermittent flow‐through respirometry, whereby water circulation alternated between a closed circuit (for measurement) and an open circuit (for flushing). The system consisted of eight tubular glass chambers (diam = 1.5 cm, length = 4 cm; volume = 6 mL) immersed in a 10‐L tank. One control chamber was left empty to monitor background levels of microbial respiration. An automated, computer‐controlled system driven by AutoResp^TM^ software (Loligo Systems, Viborg, Denmark) simultaneously measured oxygen consumption in the chambers and controlled flushing and recirculating pumps. The measurement cycle consisted of a 10‐min flushing period, a 2‐min waiting period, and a 8‐min measurement period. This ensured that the *R* ^2^ for the oxygen consumption slope exceeded 0.9, and that oxygen content in the chambers never fell below 85% during the measurement phase. Constant temperature (25.1, range = ±0.1°C) was maintained throughout the experiment by keeping the room cool and then using a computer‐controlled pump that cycled the water into a heat exchanger immersed in a warm water bath. Fish were exposed to ambient light conditions (~10 h light, 14 h dark).

Oxygen partial pressure was quantified using fiber optic probes connected to OXY‐4 oxygen meters (Presens, Regensburg, Germany), integrated into the automated system. Measurements were taken every second during the 8‐min measurement phase. The slope of the declining oxygen content in chambers (determined by linear regression) was used to calculate whole‐animal metabolic rate using the following equation:

$$V_{O_{2}}=K\times V$$

where $V_{O_{2}}$ = oxygen consumption rate (mg O_2_/h), *k* is the slope of the oxygen consumption over time, and *V* is the respirometric volume. As suggested for eco‐physiologists, we used whole‐animal metabolic rate rather than mass‐specific metabolic rate, and then accounted for mass in data analyses (Hayes 2001; Lighton 2008). From these respirometry trials, we extracted two measures of resting metabolism, the lowest value observed and the lowermost 10% of all values. We also extracted two measures of peak metabolism (that may reflect stress and attempts to escape), the average of the first five measurements (1.5 h) during the trial and the uppermost 10% of all values. The overall average RMR (as the minimum value) was 20.2* *mg O_2_/h (CV = 19) and the average peak was 35.6* *mg O_2_/h (CV = 33).

### Data analysis

Data were analyzed using a multivariate mixed model (Proc Mixed, SAS 9.2, Cary, NC); (Littell et al. 2006). Trait values were first ln‐transformed to normalize the data and then standardized (*z*‐score) to facilitate the multivariate analyses because trait variances differed substantially. The model included activity, displays, latency to feed, minimum (resting) metabolism, and initial peak metabolism as dependent variables. In order to estimate the among‐individual variance for each trait, and covariances between traits, we fitted a random intercept with respect to individual identity for each trait. We attempted to include all four metabolism measures outlined above in the same model, but the very high correlation between the different measures and complexity prevented model convergence. We therefore included only RMR and initial peak MR. We also ran the same model but instead used the upper‐ and lower‐most 10% values as our metabolism measures; this yielded near‐identical results, and so those results are not presented. Mass was included as a fixed effect for each trait to account for the expected size dependency of metabolic rate and also to assess any size dependency of behavior. Day was also included as a fixed effect for all traits in order to capture any temporal trends in our longitudinal data. A separate residual variance was fit to each trait. All fixed and random terms were retained in this model, and no model culling was performed. Within‐individual residual covariance parameters in our multivariate model were constrained to 0 because trait measurements were not matched in time. To evaluate the significance of (co‐)variance estimates, we used the “covtest” option, which yields a z‐test and provides a somewhat conservative test relative to one that assumes a variance parameter is bounded on zero. Trait repeatability was calculated as the variance of a given trait, divided by the sum of that variance and its residual variance (see Results for an example).

A preliminary and separate univariate analysis of metabolic rate measures indicated no evidence of any individual‐specific temporal trends across trials using random regression, and mass and day as fixed effects. Thus, we did not include random slope effects with respect to time in our final model to reduce model complexity (multivariate models with these effects did not converge).

## Results

### Mean‐level effects

Several traits were significantly affected by mass. Of the three measures of behavior, only activity rates were related to mass, whereby larger individuals had lower activity rates; as expected, fish that were heavier also had substantially higher metabolic rates than smaller ones (all three coefficients *P* < 0.05; Table 1). Several traits were also significantly affected by day. Fish increased activity rates and became more voracious (lower latency to feed) over time (both coefficients, *P* < 0.05; Table 1), but display intensity did not vary over time. Initial peak metabolism did not vary across days (both *P* > 0.1), whereas minimum metabolic rate (RMR) declined over time (*P* < 0.02; Table 1).

**Table 1:** Parameter estimates and associated statistics for the mean‐level effects in the multivariate mixed model

Effect	Trait	Estimate	SE	df	*t* value	*P*
Intercept	Activity	0.86	0.62	136	1.4	0.165
Intercept	Display	1.19	0.90	136	1.32	0.190
Intercept	Feeding	0.20	0.65	136	0.3	0.763
Intercept	peakMR	−2.62	0.64	136	−4.1	<0.0001
Intercept	RMR	−3.71	0.35	136	−10.73	<0.0001
Mass × trait	Activity	−11.25	5.42	793	−2.08	0.038
Mass × trait	Display	−10.92	7.24	793	−1.51	0.132
Mass × trait	Feeding	2.37	5.73	793	0.41	0.680
Mass × trait	peakMR	**24.69**	5.68	793	4.35	<0.0001
Mass × trait	RMR	**37.19**	2.90	793	12.82	<0.0001
Day × trait	Activity	**0.060**	0.012	793	4.96	<0.0001
Day × trait	Display	0.000051	0.013	793	0	0.997
Day × trait	Feeding	−**0.070**	0.012	793	−5.9	<0.0001
Day × trait	peakMR	−0.004	0.005	793	−0.75	0.456
Day × trait	RMR	−**0.011**	0.004	793	−2.83	0.005

Bold values indicate significant slope coefficients.

### Individual‐level effects

After accounting for the mean‐level effects of time and mass, we observed consistent individual differences in all traits except for minimum MR (RMR). That is, among‐individual variance and thus repeatability (*R*) was significant (*P* < 0.005) for all traits except RMR. Activity (*R *=* *0.33), display intensity (*R *=* *0.51), and voracity (*R *=* *0.36) had low‐to‐moderate repeatability; repeatability of RMR was very low and not significant (*R *=* *0.06), whereas repeatability of peak MR was significant (*R *=* *0.42). The variances and residual variances used to calculate the repeatability of each trait are presented in Table 2b (for example, *R*_activity_ = 0.303/(0.303 + 0.617) = 0.33). In addition, we provide credible intervals for repeatability estimates obtained from separate models considering each trait separately using MCMC methods (Table 2c).

**Table 2:** (a) Among‐individual correlations across behavioral and metabolic traits. (b) variance and covariance parameter estimates upon which these correlations (and repeatability values) are based. Variance estimates in the matrix of random effects are identified under “group,” while all other elements are covariances. Estimates in bold type indicate those that were significant; corresponding *z*‐tests, se, and *P*‐values are given for each. Note that non‐significant correlations nearing or exceeding 1.0 can arise when one of the variance components comprising the covariance nears zero as is the case with RMR. (c) Repeatability estimates and credible intervals generated using the rpt package of R, using MCMC methods; each was generated using a separate (univariate) model for each trait

(a) Among‐individual correlations					
Trait	Activity	Display	Feeding	PeakMR	RMR
1 Activity	1				
2 Display	−0.114	1			
3 Feeding	−**0.685**	−0.008	1		
4 PeakMR	−0.060	−**0.566**	−0.021	1	
5 RMR	−0.400	−1.000	−0.077	0.937	1

(b) Variance and covariance parameter estimates in matrix of random effects					
Matrix element	Group	Estimate	SE	*Z* Value	Pr > *Z*
(1,1)	Activity	**0.303**	0.098	3.1	0.001
(2,1)		−0.045	0.102	−0.44	0.6568
(2,2)	Display	**0.520**	0.201	2.59	0.0048
(3,1)		−**0.222**	0.084	−2.64	0.0083
(3,2)		−0.004	0.122	−0.03	0.9766
(3,3)	Feeding	**0.347**	0.109	3.18	0.0007
(4,1)		−0.018	0.078	−0.23	0.8142
(4,2)		−**0.227**	0.114	−2.00	0.0455
(4,3)		−0.007	0.086	−0.08	0.9368
(4,4)	PeakMR	**0.310**	0.118	2.63	0.0043
(5,1)		−0.031	0.040	−0.76	0.4446
(5,2)		−0.128	0.079	−1.62	0.1045
(5,3)		−0.006	0.046	−0.14	0.8899
(5,4)		0.073	0.045	1.61	0.1075
(5,5)	RMR	0.020	0.048	0.41	0.3424
Residual	Activity	0.617	0.053	11.6	<0.0001
Residual	Display	0.494	0.064	7.76	<0.0001
Residual	Feeding	0.605	0.052	11.58	<0.0001
Residual	PeakMR	0.432	0.072	5.99	<0.0001
Residual	RMR	0.324	0.062	5.24	<0.0001

(c) Trait repeatability estimates and credible intervals determined from separate MCMC runs					
Trait	*R*	2.50%	97.50%		
Activity	0.36	0.22	0.54		
Display	0.51	0.32	0.72		
Feeding	0.40	0.25	0.58		
RMR	0.15	0.03	0.36		
peakMR	0.36	0.16	0.59		

**Figure 2:**
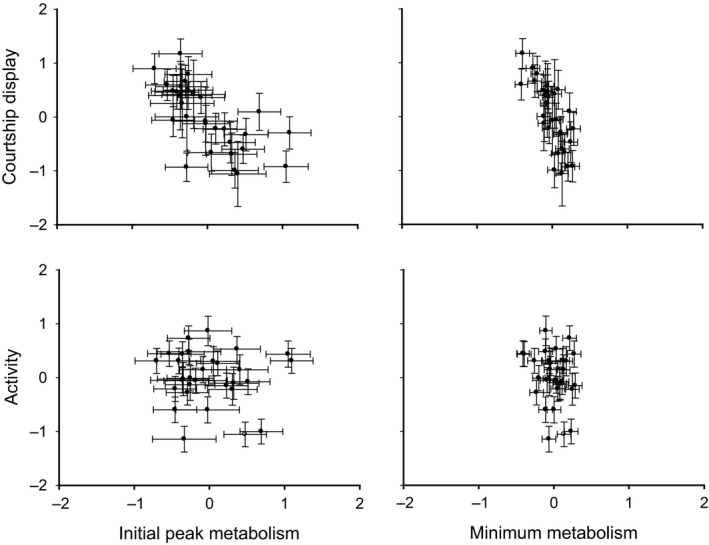
Among‐individual correlations between measures of resting (RMR) and peak metabolic rate (peak MR) in relation to two behavioral traits thought to differ in energetic demand. Shown are the model predicted values (i.e., the BLUPs and their SE's) extracted from the mixed model.

**Figure 3:**
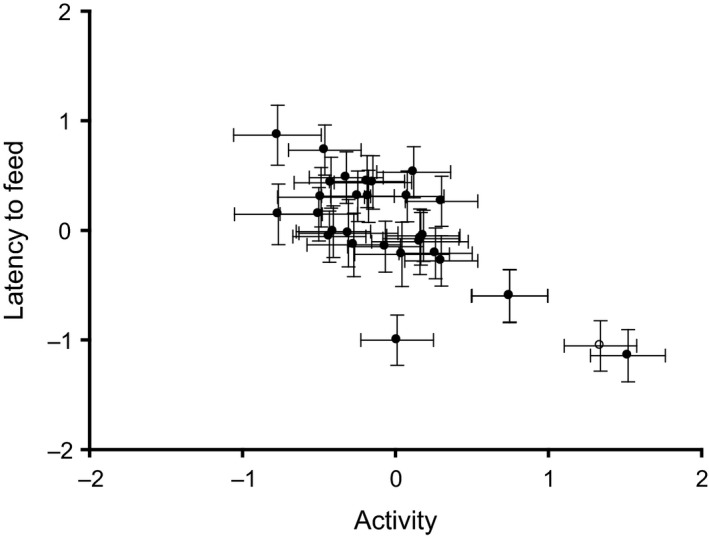
Among‐individual correlations between latency to feed (a measure of voracity) and activity. Model predicted values derived as described in Figure 2.

Among individuals, those with the highest initial peak MR were those that had significantly lower display intensity (*r *=* *−0.57), but other correlations between metabolism and activity or courtship displays were not significant (Fig. 2; see also Table 2a). Among the behavioral traits, individuals expressing higher levels of activity on average were also more voracious (i.e., had shorter latency to feed: *r *=* *−0.68; Fig. 3). All remaining among‐individual correlations (covariances) between traits were not significant (Table 2a,b).

## Discussion

We predicted that if metabolic rate is a proximate mechanism underlying behavioral variation, then individuals that were generally more active and displayed to females more intensely would also have higher RMR and be more voracious, under the assumption that RMR reflects energetic capacity (Careau et al. 2008; Biro and Stamps 2010). Contrary to these predictions, we observed that RMR was not repeatable, meaning that individuals did not differ in RMR and therefore no covariance between RMR and behavior should be observed (and indeed none were observed). By contrast, peak MR (related to stress and/or attempts to escape; see Careau et al. 2008), observed just after placing fish in respirometry chambers, was negatively correlated with display intensity but not related to activity. However, we did observe that more active individuals were also more voracious, but there was no correlation between activity and display intensity, and no correlation between displays and voracity.

We were surprised to find that measures of RMR were not repeatable, given that significant (but relatively low) repeatability of this trait is a common observation in many animals (see below). Given that we did not detect any consistent individual differences in RMR, among‐individual correlations with other traits cannot exist. It is unlikely that lack of repeatability of RMR in our study was due to low statistical power given our sample sizes (see Wolak et al. 2012), especially relative to previous studies which found significant repeatability (Nespolo and Franco 2007; Norin and Malte 2011; White et al. 2013). Rather, among‐individual variance was low (relative to residual variation) for reasons that we could only speculate upon. We did observe that individuals exhibited habituation to the respirometry trials, as evidenced by the mean‐level decline in RMR (but not peak MR) over time. We tested for individual differences in rate of apparent habituation of RMR using univariate random regression, but found no evidence of it. Of course, power for this last analysis is low given our sample size (van de Pol 2012). If individual differences in the rate of habituation were in fact present, but went undetected, then this could explain the lack of repeatability because this source of variation would increase error variance (discussed by Biro and Stamps 2015). Finally, it is possible that these fish required even more time to habituate, given that the difference between RMR and peakMR (20.2 vs. 35.6 mg/O_2_/h) was fairly small; in other words, it is possible that high levels of stress caused among‐individual variance to be reduced.

By contrast to RMR, initial peak MR, reflecting stress and movement shortly after being placed into confinement, was repeatable. This suggests that peak metabolism might be a valid measure of stress response in guppies and highlights the value of considering measures of metabolism other than just RMR that can be extracted from a given respirometry trial (as suggested by Careau et al. 2008). The negative correlation observed between peak metabolism and display intensity would then suggest that individuals that are less prone to effects of stress are also more likely to invest heavily in courtship behavior and mating. Such relationships between stress responsiveness and mating have been previously recorded in other species (reviewed in Ducrest et al. 2008) and are in agreement with general theory and empirical data on stress responsiveness in the coping styles literature (e.g. Koolhaas et al. 1999).

All of the behavioral traits were repeatable, and of a magnitude similar to previous studies (Bell et al. 2009). However, we observed the expected correlations indicating that the behavioral traits and underlying metabolic variation are not linked together in ways predicted by recent theoretical advances (Biro and Stamps 2008, 2010; Sih and Bell 2008; Réale et al. 2010; Burton et al. 2011). Our observation that higher spontaneous activity in home tanks was associated with greater voracity suggests that activity is supported by concurrent energy intake, under the assumption that latency to feed reflects hunger and intake rate. If our estimates of activity reflect overall daily levels of activity, then we could reasonably infer that active individuals have generally higher energy expenditure levels which are not supported by higher RMR. Perhaps levels of activity in home tank environments with abundant food does not need to be supported by high metabolism, or perhaps active individuals allocate less energy to maintenance (i.e., a trade‐off across individuals; discussed by Careau et al. 2008). In contrast, a lack of correlation between display intensity and voracity is perhaps not surprising given that mating displays in our experimental setting were not sustained over time on a regular basis (unlike in normal conditions where females are always present). The absence of high levels of behavioral activity displaying to and chasing females may have resulted in downregulation of resting metabolism during the weeks when males were held in isolated home tanks, and if this reduction was greater for males with higher RMR, then this might explain the low among‐individual variation in RMR. Additionally, there was no correlation between activity and display intensity, indicating little support for the notion of a “behavioral syndrome” or a general “pace‐of‐life syndrome” in this species under our laboratory conditions (Biro and Stamps 2010; Réale et al. 2010).

Apart from the individual‐level results, there were several mean‐level trends observed that suggested habituation over time despite the fact that fish were acclimated to home tanks for 1 week prior to the first behavioral measures. Activity and voracity both increased over time similarly for all individuals, but activity was lower for heavier males. Low activity and low feeding motivation are both signs of stress in fish, suggesting continued habituation over the 10 days of observations even after a week of prior acclimation. Similarly, we would expect elevated metabolism for animals under stress (Careau et al. 2008), and so it is perhaps not surprising we observed declines in resting (but not peak) MR across successive trials.

A limitation of our study, apart from a fairly small sample of males, is the fact that we did not concurrently measure all traits over time and the fact that males were not routinely exposed to females on a regular basis even outside assay times. Concurrent sampling would have allowed us to test for within‐ and as well as across‐individual correlations between behavior and metabolism which has the potential to reveal within‐individual trade‐offs between energy allocations among competing demands. Routinely exposing males to females throughout would also have prevented or reduced the potential for downregulation of metabolism, particularly important if individuals differ in their capacity to up‐ and downregulate metabolism to changing conditions.

In conclusion, while we observed significant repeatability for most traits in our study, few were correlated with each other among individuals. While repeatability estimates provide some information about heritability of traits, our study suggests that most behavioral and metabolic traits are largely independent of one another and thus their independent evolution is potentially unconstrained.

## Conflict of Interest

None declared.
